# Minimal Invasive Thoracoscopic Mitral Valve Surgery

**DOI:** 10.21470/1678-9741-2020-0260

**Published:** 2022

**Authors:** Liurong Cheng, Hongying Zhu, Wenzhen Xing, Min Fu, Yajuan Ke

**Affiliations:** 1 Hainan General Hospital, Hainan Province, China; 2 Hainan Affiliated Hospital of Hainan Medical University, China

**Keywords:** Heart Valve Diseases, Femoral Artery, Prostheses and Implants, Cardiopulmonary Bypass, Anticoagulants, Disseminated Intravascular Coagulation, Thoracoscopy, Intensive Care Units

## Abstract

**Introduction:**

The totally thoracoscopic approach for mitral valve (MV) disease is a minimally invasive method. We investigated the procedure’s feasibility, safety and effectiveness when it was performed by an experienced operator.

**Methods:**

We retrospectively analysed 96 consecutive patients with MV disease treated between March 2016 and November 2019 by minimally invasive procedures. The procedures were performed on a femoral artery-vein bypass through two ports, including a main operation port and a thoracoscopic port. The clinical data of patients were collected, including preoperative cardiac function, operative data, postoperative complications, and follow-up.

**Results:**

A total of 96 patients (57 male patients; average age, 49.7±14.5 years; left ventricular ejection fraction, 65.6±7.7%) were enrolled in this study. No intraoperative conversion incision or death occurred. The cardiopulmonary bypass and aortic cross-clamp times were 163.8±50.6 minutes and 119.7±38.9 minutes, respectively. Postoperative chest tube drainage in the first 24 hours was 232.8±108.1 ml. The ventilation time and length of intensive care unit stay were 13.2±6.2 hours and 2.9±2.2 days, respectively. One patient died of disseminated intravascular coagulation and prosthesis thrombosis 3 days after the operation, fearing anticoagulant-related hemorrhage. The overall success rate of valve repair during 1-year follow-up was 97.9%.

**Conclusion:**

The totally thoracoscopic procedure on mitral valves by an experienced surgeon is technically feasible, safe, effective and worthy of widespread adoption in clinical practice.

**Table:** 

Abbreviations, acronyms & symbols	
CO_2_	= Carbon dioxide
CPB	= Cardiopulmonary bypass
LAAL	= Left atrial appendage ligation
MS	= Median sternotomy
MVP	= Mitral valve plasty
MV	= Mitral valve
TV	= Tricuspid valve

## INTRODUCTION

Cardiac surgery via median sternotomy (MS) as a conventional approach has its drawbacks, including inevitable blood loss and transfusion, unbearable postoperative pain, and a long period for recovery^[1]^. To improve postoperative outcomes, minimally invasive approaches, including an upper and lower incision on the sternum and left and right anterolateral incisions, have been performed^[2]^. Endeavours to reduce surgical trauma, hasten patient recovery, improve cosmetic appearance, and increase patient satisfaction continued to promote minimally invasive procedures. Additional minimally invasive surgical approaches, such as total thoracoscopic or robotic assistance, have also been applied to repair congenital heart defects to minimize surgical trauma and improve cosmetic results^[3]^.

The two-incision totally thoracoscopic approach is deployed through two small incisions, including a main operation port (4-6 cm) and a thoracoscopic visual port (2-3 cm). The performance of this approach under thoracoscopy has only rarely been previously reported^[4,5]^. A total of 96 patients have received mitral valve plasty (MVP) by the two-incision approach in Chinese PLA General Hospital since March 2016. Here we report the three-year experience of our department regarding the totally thoracoscopic mitral valve plasty performed by a single experienced surgeon.

## METHODS

### Patients

This was a single-centre, retrospective, observational study of prospectively collected data from consecutively recruited patients. This study was approved by the ethics committee of our hospital. Written informed consent was preoperatively obtained from each participant and/or their parents or guardians, and the patients were fully informed about the technique and were able to choose a standard median sternotomy according to their preference. We initiated the protocol in early 2017 and, since then, totally thoracoscopic procedure on mitral valve (MV) has become the preferred approach for selected patients with MV disease, regardless of whether the disease was isolated or combined with tricuspid valve (TV) or congenital heart disease. The selection criteria were as follows: (1) isolated MV disease and no combination with serious aortic or coronary artery disease; (2) ejection fraction ≥40% or available to the operator; (3) no previous history of right thoracotomy with expected pleural cavity adhesion; (4) preoperative pulmonary function test suggesting slight dysfunction of the lungs or normal lungs; (5) no expected difficulty in femoral vessels cannulation or vena cava occlusion; and (6) a weight above 50 kg.

### Surgical Procedure

Cardiopulmonary bypass (CPB) was instituted via femoral arterial and venous cannulation through a 2-3 cm transverse incision in the right groin. Retrograde perfusion was performed through the right femoral artery (18-24 Fr). The tip of the venous cannula was positioned in the inferior vena cava (22-28 Fr), and then a second venous cannula was inserted percutaneously through the right internal jugular vein and positioned in the superior vena cava (16-18 Fr). Patient temperature was cooled to 34 ºC, and vacuum-assisted CPB was used throughout the procedure. The surgical approach was performed via two ports in the right chest.

### Main Port

A right lateral mini-thoracotomy, about 3-4 cm long, was performed in the 4^th^ intercostal space; the specific size should be sufficient for the artificial valve to be passed through. For male patients, the incision was made just below and lateral to the nipple, and for female patients, the incision was placed in the sub-mammary crease. A small tissue retractor was utilized to protect the incision.

### Thoracoscopic Port

**Fig. 1:**
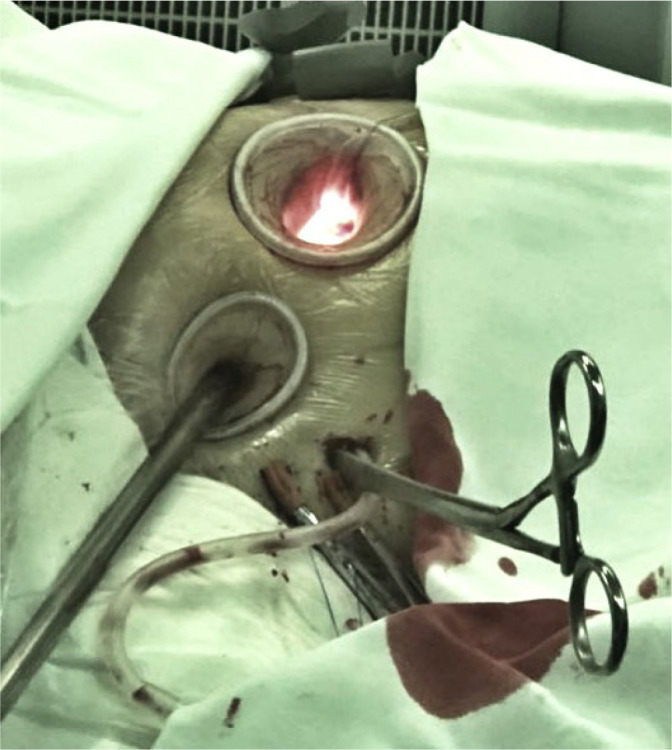
Position of the main and thoracoscopic ports.

A video camera was inserted through a 2 cm port in the 4^th^ intercostal space in the proximal midaxillary line. A transthoracic Chitwood aortic clamp and a left ventricular vent were inserted into the thoracic cavity through the next intercostal space (Figure 1).

**Fig. 2:**
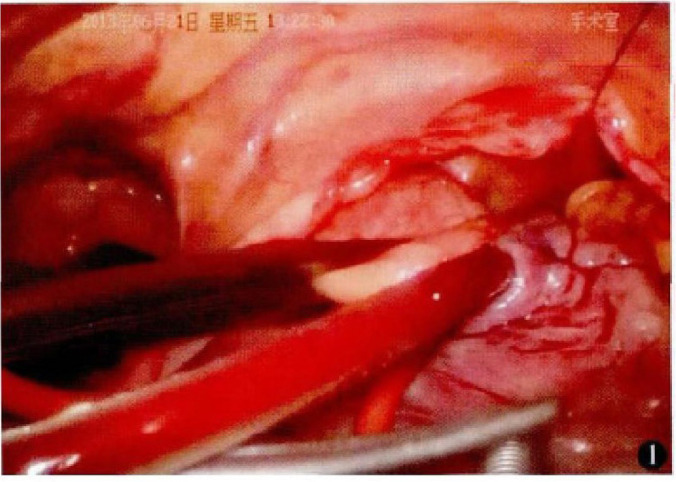
Ascending aortic clamping under thoracoscopic approach.

Antegrade crystalloid Bretschneider cardioplegia (2:l) was administered directly into the aortic root, and then continued for 90-120 minutes, if necessary. The surgical field was flooded with CO_2_ through the camera port throughout the procedure. The pericardium should be opened after the patient is placed on CPB. The left atrium was opened posteriorly to the interatrial groove. A left atrial retractor was used to expose the MV. Specialized long-shafted surgical tools were utilized for tissue handling and suturing. Standard MV repair was performed under totally thoracoscopic vision. Concomitant left atrial appendage ligation (LAAL) and other procedure could be performed routinely. Deairing was performed via a left ventricle drainage tube and the cardioplegia puncture site in the ascending aorta (Figure 2).

Intraoperative transesophageal echocardiography was used to determine the immediate results of the repair and check for perivalvular leakage and residual bubble. If the outcome was satisfactory and there was no active bleeding in the incisions, the pericardium was closed with interrupted sutures, and the incisions were sutured using the thoracoscopic approach. CPB should be stopped after the central temperature returns to normal. Two-lung ventilation was then conducted, and a thoracic drainage tube was placed through the thoracoscopic port.

### Perioperative Management

Following the operation, patients were monitored in cardiac surgical intensive care unit and were transferred to the wards as soon as they were haemodynamically stable. Additionally, chest X-rays and blood gas analyses were routinely performed to exclude complications in the lungs. Transthoracic echocardiography was performed one week after operation, 3 months later and then annually after surgery to assess the postoperative condition.

### Statistical Analysis

The short-term outcome consisted of all major adverse events, including intraoperative conversion to sternotomy, re-exploration for bleeding, valve-related reoperation within the same hospital stay, and death. Statistical analysis was performed using SPSS 25.0 software (SPSS Inc., Chicago, IL). Categorical variables were presented as frequencies and percentages, and continuous variables were presented as mean ± standard deviation.

## RESULTS

A total of 96 patients (males 59.4%, age 49.7±14.5 years) were included in this study. Table 1 shows the basic characteristics of the patients. Six patients underwent secondary cardiac surgery, of which two patients underwent MVP and the other four patients underwent congenital heart disease repair previously. Preoperative echocardiography showed that 93.8% of patients (n=90) had mitral regurgitation, of which 82.2% (n=74) were severe regurgitation. The lesions of MV regurgitation occurred mainly in A2 (16.7%, n=15) and P2 (23.3%, n=21) zones.

**Table 1:** Basic characteristics of the patients.

Baseline data	Male, n (%)		57 (59.4)
Age (years)		49.7±14.5
Hypertension, n (%)		28 (29.2)
Coronary heart disease, n (%)		13 (13.5)
Atrial fibrillation, n (%)		15 (15.6)
Infective endocarditis, n (%)		7 (7.3)
History of cerebral infarction, n (%)		10 (10.4)
Peripheral vascular disease, n (%)		5 (5.2)
Chronic obstructive pulmonary disease, n (%)		6 (6.3)
Chronic kidney disease, n (%)		4 (4.2)
Echocardiography (on admission)	Mitral regurgitation grade	III	16 (16.7)
		IV	74 (77.1)
	Mitral stenosis		6 (6.3)
	NYHA functional class III/IV, n (%)		23 (24.0)
	Redo		6 (6.3)
	Ejection fraction (%)		65.6±7.7
	Left atrium diameter (mm)		44.2±7.9
	Lesion of MV regurgitation	A1	7 (7.8)
		A2	15 (16.7)
		A3	4 (4.4)
		P1	6 (6.7)
		P2	21 (23.3)
		P3	9 (10)
		Infective endocarditis	7 (7.8)
		Commissure dislocation	6 (6.7)
		Others	15 (16.7)
Laboratory examination (on admission)	Hemoglobin (g/L)		131.2±20.8
	Alanine aminotransferase (U/L)		20.7±13.0
	Serum albumin (g/L)		41.5±4.4
	Creatinine (umol/L)		81.7±48.5
	Total bilirubin (umol/L)		14.5±5.7

All patients successfully received MVP, without intraoperative conversion or death (Table 2). Artificial chordae tendineae implantation was performed in 56.3% of patients (n=54). The overall transfusion rate was 58.3% (n=56). LAAL and resection of the left atrial myxoma were performed in 5 (5.2%) and 1 (1.0%) patient, respectively. The mean CPB and cross-clamp times were 163.8±50.6 and 119.7±38.9 minutes, respectively. The mean postoperative mechanical ventilation and ICU times were 13.2±6.2 hours and 2.9±2.2 days, respectively. The mean volume of blood drainage was 232.8±108.1 ml for the first 24 hours.

**Table 2:** Operative data and clinical outcome.

Operative data	Cardiopulmonary bypass time (min)	163.8±50.6
Cross-clamp time (min)	119.7±38.9
Intraoperative blood loss (ml)	285.3±119.4
Transfusion rate (all blood products)	56 (58.3)
Intraoperative plasma transfusion (U)	2.9±2.8
Intraoperative red blood cell transfusion (U)	1.2±1.8
Prosthesis size (mm), n (%)	25	1 (1.0)
	28	10 (10.4)
	30	32 (33.3)
	32	40 (41.7)
	34	12 (12.5)
	36	1 (10.4)
	ICU time (day)	2.9±2.2
	Post-operative time (day)	6.2±6.4
	24-hour drainage volume (ml)	232.8±108.1
	Postoperative ventilation time (hour)	13.2±6.2
Complications	Conversion to sternotomy, n (%)	0
	Reoperation for bleeding, n (%)	1 (1)
	In-hospital death, n (%)	1 (1)
	Transient neurocognitive dysfunction, n (%)	0
	Early failure requiring reoperation (<30 days), n (%)	0 (0)
Laboratory examination (1^st^ day after operation)	Hemoglobin (g/L)	111.5±17.9
	Alanine aminotransferase (U/L)	24.2±22.7
	Serum albumin (g/L)	35.7±5.4
	Creatinine (umol/L)	78.8±30.6
	Total bilirubin (umol/L)	24.6±17.6
	Creatine kinase MB (ng/ml)	26.2±22.7
Postoperative echocardiography (1 week)	Ejection fraction (%)	58.8±7.7
	Left atrium diameter (mm)	34.3±6.1

Reoperation for bleeding occurred in one (1.0%) patient. Another patient died of cardiogenic shock 3 days after operation. No early MVP failure requiring secondary surgery occurred.

The 1-year follow-up was 100% complete, with a mean follow-up time of 15.7±9.2 months. The overall success rate of valve repair during the 1-year follow-up was 97.9%. Two patients underwent valve replacement surgery due to perivalvular leakage 3 months and 7 months after the operation, respectively. No deaths, infective endocarditis, pulmonary atelectasis, or moderate tricuspid regurgitation were found.

## DISCUSSION

Although traditional median sternotomy cardiac surgery now provides good surgical vision and outcomes, it requires that the sternum be completely sawed through, which damages the sternal integrity, increases bleeding and postoperative pain, can cause the creation of a hernia under the xiphisternum or mediastinal infection, and leaves residual permanent steel wire.

In the past few decades, minimally invasive methods of cardiac surgery have developed and improved continuously, with thoracoscopic cardiac surgery technology advancing accordingly. Compared with conventional median sternotomy, the sternum-exempt procedure could not only preserve the integrity of the osseous thoracic wall, but also save time in the formidable task of haemostasis as in the median sternotomy. Besides, compared with the right intercostal small incision approach, thoracoscopic technique can create a small anterolateral incision^[6,7]^. Percutaneous mitral interventions are alternative treatment options for patients who are deemed to be at high surgical risk and/or inoperable. Transcatheter edge-to-edge mitral valve repair using the MitraClip and PASCAL system, which are designed to mimic the surgical Alfieri stitch, has changed the landscape for the treatment of symptomatic functional mitral regurgitation. The transapical off-pump mitral valve repair with neochord implantation, known as NeoChord procedure, is also a new option to implant artificial chords in a minimally invasive manner in MR patients with leaflet prolapse or flail. Transcatheter mitral valve replacement is another emerging treatment option for selected patients. However, we need to realize that the several percutaneous procedures mentioned are still in the exploratory stage. RCT experiments with long-term follow-up results are lacking to confirm the surgical effect^[8,9]^. A meta-analysis demonstrated that minimally invasive and sternotomy approaches produce comparable results for complex mitral valve repair, but the thoracoscopic technique, as a minimally invasive surgical approach, is easily selected and accepted by patients^[10]^. The result of patients who received MVP in our department revealed that this approach was technically feasible, did not require transition to median sternotomy, and had a low rate of adverse events.

But the totally thoracoscopic procedure for MV disease is still technically challenging, and its application is currently restricted to a handful of experienced operators because it entails the surgeon overcoming a lengthy learning curve^[11,12]^. The survey of surgeons who were experienced in minimally invasive MV surgery showed that 90% of the respondents believed that more than 20 cases were required to gain familiarity with the procedure^[13]^. Because of the small skin incision and small operation space in the total thoracoscopic approach, the surgical technique is more difficult, and most of the surgical operations need to be completed by the surgeon alone. Therefore, the CPB time and the aortic occlusion time of the total thoracoscopic approach for beginners is longer than the median thoracotomy approach. However, due to the small trauma, short time of hemostasis and chest closure, with the further accumulation of experience and the improvement of surgical proficiency, the total operation time of thoracoscopic MVP in our department is obviously shortened, even shorter than the median thoracotomy approach now.

In the early postoperative period, the use of a thoracoscopic port reduced the incidence of poor wound healing, reduced scarring, and increased the concealment of the incision site. Casselman et al.^[11]^ investigated 187 patients who underwent minimally invasive MV surgery and found that 98% of them were satisfied with the cosmetic outcome of the surgical incision. Among the 96 patients surveyed in this study, no early poor incision healing occurred. In previous studies, it remains controversial whether thoracoscopic cardiac surgery increases the risk of stroke and other complications compared with conventional sternotomy. We are very pleased to see that, after taking such measures as high flow of CO_2_ regulated before the left atrium, and a continuous low flow of CO_2_ used in the chest intra-operatively, the patients included in this test did not present neurological complications.

In addition, we must see that the thoracoscopic technique also has its limitations: the manipulation zone lacked a stereoscopic experience; the field of vision during the operation was narrowed down by an enlarged thoracoscope, so that the surgeon had increased difficulty during manipulation. Each knotting entailed crossing of the sutures outside of chest cavity and then pulling down the knots with the assistance of a knot pusher. Also, CPB establishment through femoral artery-vein bypass can be difficult for patients who have low body weight with smaller femoral blood vessels^[14,15]^.

## CONCLUSION

The thoracoscopic procedure for conventional MV diseases is worth being broadly advocated, and more experienced surgeons should be trained on this procedure due to its high demand in terms of manipulation skills and three-dimensional conception. In summary, the totally thoracoscopic procedure for MV disease by an experienced operator is feasible, safe, effective, and merits widespread adoption.
